# Women's Joint Decision on Contraceptive Use in Gedeo Zone, Southern Ethiopia: A Community Based Comparative Cross-Sectional Study

**DOI:** 10.1155/2017/9389072

**Published:** 2017-03-07

**Authors:** Akine Eshete, Yohannes Adissu

**Affiliations:** Department of Public Health, College of Medicine and Health Sciences, Dilla University, P.O. Box 419, Dilla, Ethiopia

## Abstract

A community based comparative cross-sectional study design was employed to assess the mutual consent of women about family planning use in urban and rural villages of Gedeo zone. Two-thirds (67.4%) of women made joint decision on contraceptive use, varying between urban (70.9%) and rural (63.4%) settings. This difference was statistically significant where women in urban setup had a 41% (AOR, 1.41; 95% CI (1.15, 2.01) added chance of making joint decision than the rural counterpart. In both settings, attitude towards contraceptive method was an independent predictor of joint contraceptive decision (AOR = 2.85) in urban and (AOR = 2.81) rural women. Contrarily, different factors were found to be associated with joint contraceptive decision in either setup. In urban, having better knowledge about contraceptive methods (AOR = 2.9) and having lower age difference (AOR = 2.2) were found to be strong predictors of joint decision on contraceptive use, while having too many children (AOR = 2.2) and paternal support (AOR = 7.1) in rural setups. Lower level of joint decision making on contraceptive use was reported in both setups. Factors associated with joint decision varied between the two setups, except for attitude towards contraceptive methods. Future family planning program should address sociocultural, knowledge and attitude factors.

## 1. Introduction

Population increase is a continuous burning issue globally, with statistical figure of 7.3 billion in 2015 which is expected to increase to 9.8 billion by 2050 [1]. In Ethiopia, 2 million people are added to the existing population annually and reached 98.1 million by 2015. The expected population of Ethiopia by mid-2030 and 2050 will be 130.5 million and 165.1 million, respectively [1, 2]. If the rate of population growths continues, it will be a threat to the nation's economy and social service [3].

In Ethiopia, contraceptive prevalence rate (CPR) slightly increased from 29% and 34% in 2011 to 42% and 39.8% in 2014 among married women across the nation and Southern Nations and Nationality People Region (SNNPR), respectively. As a result the maternal mortality ratio declined from 676/100,000 live births in 2011 to 353 per 100,000 live births in 2015 [4–10]. Women's decision on family planning use has multiple benefits to the family and community at large. Reports suggested that women's participation in family planning decision has to influence their reproductive health, overall health, and family balance [11–14]. Different studies revealed that women who are involved in any decision-making process are more likely to control their fertility by using contraceptives [9, 15–18].

Even though women have the right to decide their reproductive health issues, decisions are overruled by their respective husbands'. This has a negative influence on women's fertility behavior [19–25]. Therefore, efforts need to be made for women involvement and achievement of their desired goals in relation to birth spacing or limiting. Furthermore, the role and influence of husbands need to be taken into account when developing family planning services and programs [22, 26].

In Ethiopia, there exists a large variation on contraceptive decision-making power among different geographical locations. For example, 54.4% in Amhara region exercised mutual consent for contraceptive use, whereas in other regions mutual consent for contraceptive use was lower, such as in SNNPR (30.7%), in Gambella (12%), and in Ethiopia Somali (2%) [27]. Lower rates of joint decision were recorded in rural area in Ethiopia and Uganda [28, 29]. Women in Ethiopia have graduated to evolved method of family planning strategy, but the lack of definite statistics has urged this study to intervene in the gap with defined objectives as follows: (1) examining the differences in spousal agreement and her ability to use contraceptives in urban versus rural women; (2) examining the barrier of women's influence on joint decision-making to contraceptives use. In addition, for increasing contraceptive utilization among women of reproductive age, the barrier of women's influence on decision-making role needs to be addressed.

Hence producing information on women's decision-making power on family planning use has a paramount importance for designing appropriate program. Therefore, this study is conducted to address this through: (1) describing the current status of contraceptive use in urban versus rural women; (2) examining the differences in spousal agreement and her ability to use contraceptives in urban versus rural women; (3) examining the barriers to women's influence on joint decision-making to contraceptives use.

## 2. Methods and Materials

### 2.1. Study Settings and Participants

Community based comparative cross-sectional study design was employed in urban and rural villages of Gedeo zone. Gedeo zone has six districts and two towns, namely, Dilla and Yirgachefe. According to the 2007 census report, the total population of the zone was 1,694,868, of which 845,384 were females in all age groups [30]. Only married women who were currently living with their husbands were included in this study (Figure 4).

### 2.2. Sample and Sampling Procedure

Sample size was determined using Epi INFO version 7 considering the following assumptions: level of significance (0.05), power (0.80), proportion of having joint decision-making in urban married women (67.07%), and rural women (57.17%) [28], with consideration of design effect of 2 and the nonresponse rate of 10%. This yielded a total sample of 480 in urban and 480 in rural women.

We employed a stratified multistage sampling followed by systematic random sampling method. Initially, out of six rural districts in the zone, three districts were selected randomly by using lottery method. This was followed by random selection of six rural kebeles (smallest administration unit in Ethiopia). We also identified four urban kebeles in similar districts of the zone. The periurban kebeles in the selected districts were excluded from the study to avoid mixing of urban and rural populations. Sample size for both rural and urban areas was allocated proportionally. Census was carried out to identify households with the couples' in the selected kebeles to generate a sampling frame. Finally systematic random sampling technique was used to select the study units. If there was more than one eligible married woman in one household, one was selected randomly by using a lottery method. However, if we could not find any eligible married women in a household, we shifted to the next immediate household to the right of the index households.

### 2.3. Data Collection Methods

Data were collected with face to face interview questionnaire. The questionnaire was designed and modified in comparison with other studies [4, 28, 31]. Health extension workers were collecting the data. Four supervisors supervised the data collection process. Completed questionnaires were reviewed daily by the supervisors and principal investigators.

### 2.4. Measurement of Variables

The dependent variable was joint contraceptive decision, which was measured by asking “whom to decide to use contraceptive methods.” The response to this question was dichotomous; if the decision made by only woman, husband alone, or another person was coded as “0” and if the decision made jointly was coded as “1,” finally, contraceptive decision was dichotomous in joint and separate decisions. The other outcome of interest was current use of contraception, which was assessed by asking married women for their practice of any contraceptive method at the time of data collection.

Knowledge of contraceptive method was measured by using different questions, which was related to contraceptive methods, and having correct answers for at least 70% was considered as good knowledge [28]. Likert scale was used to measure attitude to contraceptive method. The response to each item was dichotomized and having a positive response to at least 70% of statements was considered positive attitude [28]. Gender-related variables that can serve as a proxy factor for joint decision included the interspousal age difference (husband's age minus wife's reported age).

### 2.5. Data Processing and Analysis

Data were entered and cleaned by using Epi INFO vision 3.5.1. After being cleaned by the Epi INFO it was exported to SPSS V-20 for analysis. Bivariable and multivariable logistic regression analysis was used to identify factors associated with joint contraceptive decision. Adjusted odds ratio (OR) with its 95% CI was computed to assess the strength of the association.

### 2.6. Ethical Considerations

Ethical clearance was obtained from Research and Ethics Committee of College of Health Sciences and Medicine, Dilla University. Written informed consent was obtained from each study participant. A copy of this informed consent form was provided to each participant. The information obtained would be anonymous and thereby assured of confidentiality.

## 3. Results

### 3.1. Sociodemographic Characteristics of the Respondents

The median (±SD) age of the respondents was 26 ± 5.2 and 29 ± 6.4 in urban and rural areas, respectively. In both settings, majority of the respondents were found in the age group of 25–29 years. Majority of women in urban (46.4%) and rural (35.0%) areas attended primary school. Most of the respondents in both settings were house wives. The median (±SD) monthly income was 1000 ± 1198 in urban and 736.5 ± 885.5 in rural areas (Table 1).

### 3.2. Reproductive History of the Respondents

The mean (±SD) age at first marriage was 18.5 ± 3.9 and 17.1 ± 3.3 years in urban and rural areas, respectively. In urban women, 67% had less than four children, whereas 69.1% of rural women had more than four children. One hundred and seventy-eight (33.7%) of rural women desired to have more than six children, but only 5.7% of urban women desired to have more than six children. In the same manner, 38.3% reported that their husbands desire to have more than six children in rural settings, while only 11.9% desired so in urban settings (Table 2).

### 3.3. Knowledge and Attitude towards Contraceptive Methods

In both settings, nearly equal numbers of women have ever heard about one of the contraceptive methods (97.3% in urban and 96.6% in rural). The most frequently mentioned method of contraceptive was injectable (Depo-Provera). In both settings, nearly all women knew the importance of contraceptive methods and around 48% of urban and 40% of rural women stated at least one side effect of contraceptive methods (Table 3). Overall, 70.8% of urban and 62.7% of rural women were knowledgeable about contraceptive methods. And about 76.3% of urban and 74.4% of rural women had a positive perception towards contraceptive methods.

Nearly half (53.3%) the women had discussion on contraceptive methods with their husbands in the total sample. Urban women (61.2%) had better discussion on contraceptive methods with their husbands compared to rural women (45.5%). Similarly, urban women (60.0%) received better cooperation from their husbands compared to rural women (44.9%). Around 15.9% of urban and 7.2% of rural women also got support from their relatives (Table 3).

### 3.4. Current Use of Contraception among the Study Subjects

**Figure 1:**
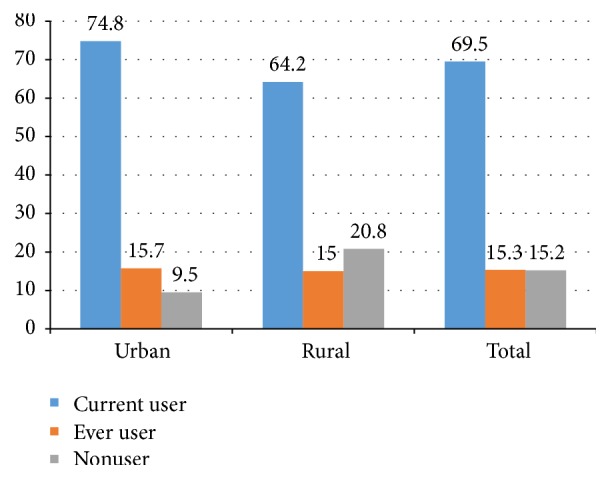
Women's group on contraceptive use in urban and rural districts of Gedeo zone, South Ethiopia, 2015.

**Figure 2:**
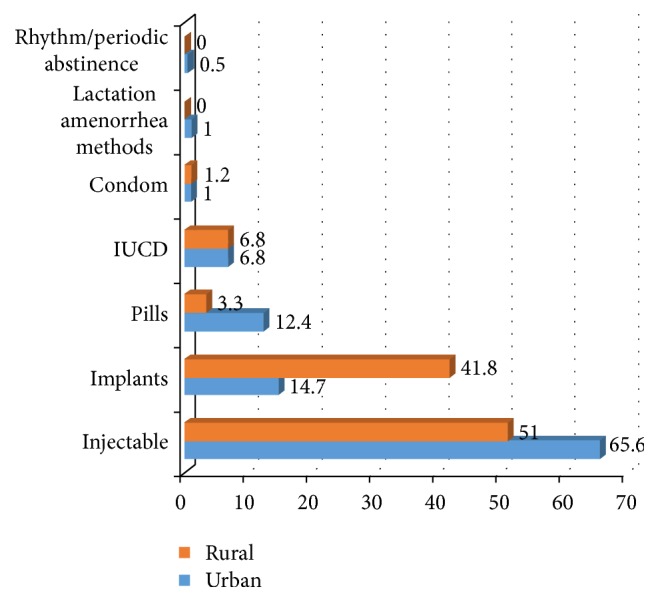
Contraceptive method used in urban and rural districts of Gedeo zone, South Ethiopia, 2015.

**Figure 3:**
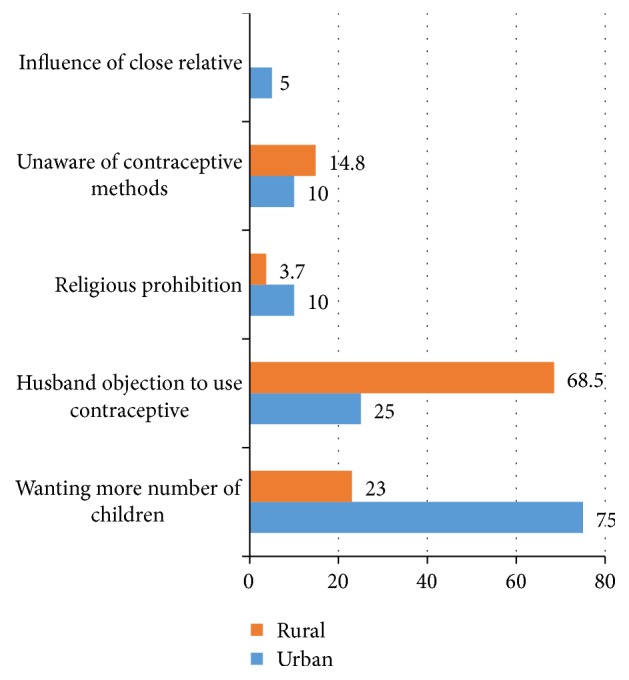
Reasons for not using contraceptive methods among married women in urban and rural districts of Gedeo zone, South Ethiopia, 2015 (n.b. Multiple responses were possible).

**Figure 4:**
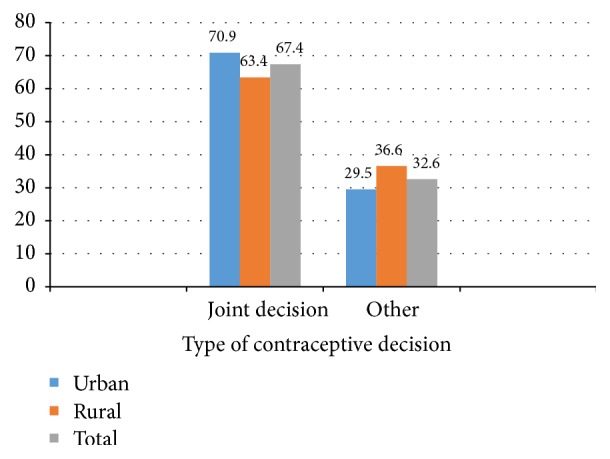
Married women's joint decision-making on contraceptive use in urban and rural districts of Gedeo zone, South Ethiopia, 2015.

In total, 734 (69.5%) were currently using contraceptives. The magnitude of contraceptive use was 74.8% in urban and 64.2% in rural areas (Figure 1). The most preferred contraceptive method in both setup was injectable (Figure 2). Nearly all current users, 389 (98.5%) of urban and 337 (99.4%) of rural women, used modern contraceptive methods. More than three-fourths, 310 (78.5%), of current users in urban setup used short term contraceptive methods, whereas 179 (52.8%) of rural current users used short term contraceptive methods. The magnitude of long acting contraceptive methods was higher in rural women, 160 (47.2%), compared to 85 (21.5%) in urban women. In both settings, the desire to have many children and husband objection were mentioned as a reason for nonuse of contraceptive methods (Figure 3).

### 3.5. Joint Contraceptive Decision-Making Power

The overall proportion of women with joint decision-making was 67.4%, varying between urban (70.9%) and rural (63.4%) settings. This difference was statistically significant where women in urban setup had a 41% (AOR, 1.41; 95% CI (1.15, 2.01)) added chance of making joint decision than the rural counterpart.

### 3.6. Factors Associated with Contraception Joint Decision-Making among the Study Participants

After controlling for possible confounders, in urban area women who had age difference less than four years were two times more likely to have joint decision on contraceptive use compared to those who had greater than four years age difference [AOR, 2.16; 95% CI (1.21, 4.89)]. Women who had more children were less likely to have joint decision on contraceptive use as compared to those who had fewer children [AOR, 0.26; 95% CI (0.13, 0.50)].

Married women who did not have a discussion on contraceptive methods were less likely to have joint decision on contraceptive use compared to their counterpart [AOR, 0.03; 95% CI (0.013, 0.089)]. And also women who received support from their relatives were less likely to have joint decision on contraceptive use as compared to their counterpart [AOR, 0.28; 95% CI (0.13, 0.58)]. However, urban women who were knowledgeable about family planning methods [AOR, 2.85; 95% CI (1.30, 6.24)] and who had positive attitudes towards family planning methods [AOR, 2.85; 95% CI (1.30, 6.24)] were more likely to have joint contraceptive decision (Table 4).

However, among rural respondents, women who had more children [AOR, 2.22; 95% CI (1.12, 4.44)], who received support from their relatives [AOR, 7.10′ 95% CI (2.32, 21.69)], and who had positive attitudes towards contraceptive methods [AOR 2.81, 95% CI (1.02, 7.70)] were more likely to have joint contraceptive decision. Moreover, women who did not have a discussion on contraceptive methods [AOR, 0.01; 95% CI (0.01, 0.023)] and who were knowledgeable about contraceptive methods [AOR 0.35, 95% CI (0.16, 0.77)] were less likely to have joint decision on contraceptive use (Table 4).

## 4. Discussion

The magnitude of contraceptive use was higher in urban compared to rural women. This result was in line with the 2011 Ethiopian Demographic Health Survey report and a study conducted in Southern Ethiopia [4, 28]. The urban rural variation may be due to the fact that, in most parts of rural Ethiopia, women usually attained low education, low socioeconomic status, and low involvement in their healthcare decisions. On the other hand, the desire to have many children and culturally influenced beliefs favoring high fertility is still a major factor for affecting the uptake of family planning methods.

Even though the study populations acknowledged the benefit of family planning methods, their utilization was influenced by many factors. In both settings, the desire to have many children and husband objection were mentioned as a reason for nonuse and it remains subordinate to the powerful social norm of having many children. This indicates that women would be forced to bear a large number of children. In addition, the survey indicated that ideal family size is high, which indicates lack of awareness about the benefit of having small family size. It is a known tradition in Ethiopia, particularly rural area; traditional cultures place a premium on large families.

In this current study, 67.4% of women made joint decision on contraceptive use in the total population. This finding was consistent with a study done in Zambia (65.8%) [17], but it was lower than the study finding in Mozambique, 71.6% [26]. Having joint decision on contraceptive use was higher in urban women (70.9%) compared to rural women (63.4%). This may be due to the fact that, in rural area, husbands desired more children and this negatively influenced a women's involvement to contraception decision.

In both settings, joint decision-making power was lower than previous studies conducted in Ethiopia, 92.4% in urban and 91% in rural women [28], and other studies in rural communities of southeast Ethiopia, 83.4% [32], in Wolaita Soddo town, 71.4% [33], in Hossana Town, 81% [34], and in the southeastern zone of Tigray region, 78% [35]. The reason was that only half, 53.3%, of the respondents had discussion on contraceptive methods with their husbands in this study. This suggests that there is a need of increasing spousal communication on family planning method in both settings. Therefore, healthcare providers and community leaders need to disseminate clear and culturally appropriate messages that will help women and their partners for common understanding of contraception.

Despite having joint contraceptive decision among participants, half (52.5%) of women received support from their husbands to use contraceptive, of which there were 60.0% in urban and 44.9% in rural communities. This implies that gender equality needs to become well engrained. Therefore, policies should be formulated to recognize woman's ability to negotiate with her husband's regarding involvement in decision-making within the household, including decisions related to her fertility.

This study attempted to identify factors associated with woman's joint decision-making position related to contraceptive use. In urban area, having better knowledge about contraceptive methods, having a better attitude towards contraceptive methods, and having lower age difference were strongly associated with joint contraceptive decision. However, getting support from their relative and number of alive children did not show strong association with joint contraceptive decision.

However, in rural areas, attitude towards contraceptive methods, number of alive children, and who received support from their relative were found were found to be strongly associated with joint contraceptive decision. Unlike urban women, rural women who had support from their relative were more likely to have joint decision on contraceptive use.

### 4.1. Limitation of the Study

The status of male partner involvement and support was indirectly obtained from woman's perspective. The impact of gender norms and sociocultural factors was not assessed in detail. More researches should be done to identify how gender myths and specific roles and power inequalities can function as a barrier to make joint decision.

## 5. Conclusions and Program Implications

In this current study, 67.4% of women made joint decision on contraceptive use in the total population. Making joint decision on contraceptive use was 70.9% in urban and 63.4% in rural women. There were lower levels of joint decision-making on contraceptive use in both setups. Factors associated with joint decision-making varied between the two setups, except for attitude towards contraceptive use.

Future family planning program should address sociocultural, knowledge and attitude factors. In addition, the role and influence of husbands need to be taken into account when developing family planning services. Furthermore, this study suggests that there is a need of increasing spousal communication on family planning methods. Lastly, family planning interventions in the area need to be promoted and considered empowerment of women on contraceptive decision-making.

## Figures and Tables

**Table 1:** Sociodemographic variables of married women in reproductive age in urban and rural districts of Gedeo zone, South Ethiopia, 2015.

Variable	Urban (%) (*n* = 528)	Rural (%) (*n* = 528)	*p* values
*Age of respondents *	Mean age, 26 ± 5.2 SD	30 ± 6.4 SD	<0.001
15–19	27 (5.1%)	13 (2.5%)	
20–24	153 (29.0%)	87 (16.5%)	
25–29	201 (29.0%)	167 (31.6%)	
30–34	91 (17.2%)	121 (22.9%)	
35–39	51 (9.7%)	82 (15.5%)	
40–44	5 (0.9%)	48 (9.1%)	
45–49	—	10 (1.9%)	
*Religion of respondents *			0.020
Orthodox	303 (57.4%)	126 (23.9%)	
Protestant	159 (30.1%)	359 (68.0%)	
Catholic	13 (2.5%)	8 (1.5%)	
Muslim	53 (10.0%)	27 (5.1%)	
Other (Pagan, Tsege)	—	8 (1.5%)	
*Ethnicity of respondents *			0.003
Gedeo	78 (14.8%)	385 (72.9%)	
Sidama	45 (8.5%)	33 (6.2%)	
Gurage	116 (22.0%)	13 (2.5%)	
Wolayita	65 (12.3%)	10 (1.9%)	
Amhara	134 (25.4%)	47 (6.8%)	
Oromo	81 (15.3%)	36 (6.8%)	
Other (Gamao Goffa, Tigre)	9 (1.7%)	4 (0.8%)	
*Educational status *			<0.001
Unable to read and write	54 (10.2%)	203 (38.0%)	
Read and write only	17 (3.2%)	66 (12.5%)	
Primary school (1–8)	245 (46.4%)	185 (35.0%)	
High school (9–12)	166 (31.4%)	50 (9.5%)	
Diploma and above	46 (8.7%)	24 (4.5%)	
*Occupational status *			0.006
Housewife	370 (70.1%)	217 (41.1%)	
Government employee	41 (7.8%)	40 (7.6%)	
Private employee	115 (21.8%)	106 (20.1%)	
Daily worker	1 (0.2%)	147 (27.8%)	
Unemployed	1 (0.2%)	18 (3.4%)	
*Income level of the household*	Median, 1000 ± 1198 SD	736.5 (±885.5 SD)	<0.001
Low income level	173 (32.8%)	368 (69.7%)	
Medium income level	220 (41.7%)	99 (18.8%)	
High income level	135 (25.6%)	61 (11.6%)	

**Table 2:** Reproductive history of married women in reproductive age in urban and rural districts of Gedeo zone, South Ethiopia, 2015.

Variable	Urban (%) (*n* = 528)	Rural (%) (*n* = 528)
*Wife Age at marriage*	Mean, 18.5 ± 3.99 SD	Mean, 17.1 ± 3.3 SD
Less than 18 years	291 (55.1%)	388 (73.5%)
18–20 years	105 (19.9%)	81 (15.3%)
Greater than 20 years	132 (19.9%)	59 (11.2%)
*Number of alive children *		
Having less than 4 children	354 (67.0%)	163 (30.9%)
Having greater than 4 children	174 (33.0%)	365 (69.1%)
*Woman's desire of family size*		
1–4 children	381 (72.2%)	150 (28.4%)
4–6 children	117 (22.2%)	200 (37.9%)
Greater than 6 children	30 (5.7%)	178 (33.7%)
*Husband's desire of family size*		
1–4 children	353 (66.9%)	149 (28.2%)
4–6 children	112 (21.2%)	177 (33.5%)
Greater than 6 children	63 (11.9%)	202 (38.3%)
*Child death experience of woman*		
Yes	47 (8.9%)	159 (30.1%)
No	481 (91.1%)	369 (69.9%)
*Number of child deaths *		
1-2 children	42 (89.4%)	120 (75.5%)
2–4 children	5 (10.6%)	37 (23.3%)
Greater than 4 children	—	2 (1.3%)

**Table 3:** Knowledge of Married women on contraceptive method in urban and rural districts of Gedeo zone, South Ethiopia, 2015.

Variables	Urban (%)	Rural (%)
*Ever heard of contraceptive methods*		
Yes	514 (97.3%)	510 (96.6%)
No	14 (2.7%)	18 (3.4%)
*Type of methods women know *	*(n = 514)*	*(n* = *510)*
Injectables	497 (97.1%)	496 (97.3%)
Pills	481 (93.9%)	418 (82.0%)
Implants	390 (76.2%)	416 (81.6%)
IUCD	386 (75.4%)	205 (40.2%)
Lactation amenorrhea methods	43 (8.4%)	9 (1.8%)
Condom	244 (47.7%)	41 (8.0%)
Rhythm/periodic abstinence	94 (18.4%)	10 (2.0%)
Permanent method	40 (7.8%)	16 (3.1%)
*Source of contraceptive method*	*(n = 514)*	*(n* = *510)*
Health professionals	430 (85.5%)	452 (93.0%)
Radio/TV	294 (58.4%)	61 (12.6%)
Neighboring	82 (16.3%)	229 (47.1%)
Husband	104 (20.7%)	48 (9.9%)
Poster/leaflet	74 (14.7%)	12 (2.5%)
*Ever known the importance of FP *	*(n = 514)*	*(n* = *510)*
Yes	509 (99.0%)	471 (92.4%)
No	5 (1.0%)	39 (7.6%)
*Importance of FP mentioned by women*		
(1) To avoid pregnancy	500 (98.8%)	382 (81.3%)
(2) To limit number of pregnancies	287 (56.7%)	401 (85.3%)
(3) To space the child	238 (47.0%)	116 (24.7%)
*Ever known side effects of contraceptives*		
Yes	246 (47.9%)	206 (40.4%)
No	268 (52.2%)	304 (59.6%)
*Side effect of FP mentioned by women*		
Weight gain	161 (85.6%)	49(24.9%)
Headache	110 (58.5%)	59 (29.9%)
Irregular menstruation	98 (52.1%)	154 (78.2%)
Vomiting	83 (44.1%)	21 (10.7%)
Nausea	79 (42.0%)	47 (23.9%)
*Women's discussion on contraceptive methods with their husbands *		
Yes	323 (61.2%)	240 (45.5%)
No	205 (38.8%)	288 (54.5%)
*Women's support from their husbands to use or not use *		
Yes	317 (60.0%)	237 (44.9%)
No	211 (40.0%)	291 (55.1%)
*Support from their close relatives *		
Yes	84 (15.9%)	38 (7.2%)
No	444 (84.1%)	490 (92.8%)

**Table 4:** Factors contributing to contraceptive joint decision making in urban and rural districts of Gedeo zone, South Ethiopia, 2015.

Variables	Urban areas		Rural areas	
	COR (95.0% CI)	AOR (95.0% CI)	COR (95.0% CI)	AOR (95.0% CI)
*Age difference *				
Less than four years	0.47 (0.27, 0.83)	2.16 (1.21, 4.89)	—	—
Greater than four years	Ref	Ref	—	—
*Number of children women have *				
Having 0–2 children	Ref	Ref	Ref	Ref
Having more than two children	0.22 (0.14, 0.35)	0.26 (0.13, 0.50)	0.58 (0.35, 0.96)	2.22 (1.12, 4.44 )
*Discussion of FP methods with their husbands*				
Yes	Ref	Ref	Ref	Ref
No	0.016 (0.01, 0.04)	0.03 (0.013, 0.09)	0.04 (0.02, 0.07)	0.01 (0.01, 0.023)
*Support from close relatives*				
Yes	7.65 (3.13, 18.67)	0.28 (0.13, 0.58)	0.77 (0.39, 1.53)	7.10 (2.32, 21.69)
No	Ref	Ref	Ref	Ref
*Knowledgeable about contraceptive methods*				
Lowly knowledgeable about contraceptive methods	Ref	Ref	Ref	Ref
Highly knowledgeable about contraceptive method	2.52 (1.60, 4.0)	2.85 (1.47, 5.51)	0.74 (0.45, 1.22)	0.35 (0.16, 0.77)
*Attitude towards contraceptive methods*				
Having wrong attitude towards contraceptive methods	Ref	Ref	Ref	Ref
Having a positive attitude towards contraceptive methods	3.76 (2.24, 6.30)	2.85 (1.30, 6.24)	0.76 (0.32, 1.79)	2.81 (1.02, 7.70)

## Abbreviations

AOR: Adjusted odds ratio
EDHS: Ethiopian Demographic Health Survey
OR: Odds ratio
SNNPR: Southern Nations and Nationality People Region.
